# Biotechnology of Cold-Active Proteases

**DOI:** 10.3390/biology2020755

**Published:** 2013-05-03

**Authors:** Swati Joshi, Tulasi Satyanarayana

**Affiliations:** Department of Microbiology, University of Delhi South Campus, New Delhi 110021, India; E-Mail: swati.joshi.aj@gmail.com

**Keywords:** Extremozymes, psychrozymes, cold-active protease, metagenomics, site directed mutagenesis, x-ray crystallography

## Abstract

The bulk of Earth’s biosphere is cold (<5 °C) and inhabited by psychrophiles. Biocatalysts from psychrophilic organisms (psychrozymes) have attracted attention because of their application in the ongoing efforts to decrease energy consumption. Proteinases as a class represent the largest category of industrial enzymes. There has been an emphasis on employing cold-active proteases in detergents because this allows laundry operations at ambient temperatures. Proteases have been used in environmental bioremediation, food industry and molecular biology. In view of the present limited understanding and availability of cold-active proteases with diverse characteristics, it is essential to explore Earth’s surface more in search of an ideal cold-active protease. The understanding of molecular and mechanistic details of these proteases will open up new avenues to tailor proteases with the desired properties. A detailed account of the developments in the production and applications of cold-active proteases is presented in this review.

## 1. Introduction

Life exists at temperatures as low as −20 °C in the permafrost soil and as high as 122 °C in thermal environments [1,2]. Almost 70% of our planet’s surface is covered by oceans, and thus, is a major environment where the temperature is around 4 °C. Polar regions constitute 15% of the Earth’s surface and 20% of the terrestrial region of Earth is permafrost. Thus 80% of earth’s surface is permanently cold with the temperatures below 5 °C [3]. All the cold geographical regions of the Earth harbor cold-adapted microorganisms, which are known as psychrophiles [4]. Modern biotech industry requires macromolecules that can function under extreme conditions. Psychrophilic and psychrotolerant microorganisms and their cold-adapted proteins and enzymes have a host of biotechnological applications [5]. Microorganisms get adapted to different niches, and thus, lead to evolution in their molecular machinery. The cold-adapted microbes are known to produce cold-active enzymes [6]. Among cold-active enzymes (α-amylase [7,8], lipase [9,10], aspartate transcarbamylase [11], Ca^+^Zn^+2^ protease, [12], citrate synthetase, [13], α-lactamase [14], malate dehydrogenase [15], triose-phosphate isomerase [16], DNA ligase [17], xylanase [18], citrate synthase [19], metalloprotease [12], polygalacturonase [20], cellulases and xylanase [21], chitinase [22], endo-arabinanase [23], and pectinase [24]), proteases constitute an important group which have high catalytic efficiencies at lower temperatures. Proteases constitute an important class of hydrolytic enzymes that are found in all life forms as they are essential in physiological, metabolic and regulatory functions [25]. Nowadays, approximately 60% of the total enzyme market is shared by proteases in various industries, and according to a recent report from Business Communications Company (BCC 2008), the global market for industrial enzymes had been estimated to reach US $ 4.9 billion by 2013 [26,27]. Proteases have found applications in diverse fields such as detergent industry, leather processing, silk degumming, food and dairy, baking, pharmaceutical industries, silver recovery from x-ray films, waste management and others. Cold-active proteases have been reported from various microorganisms, but detailed investigations on their adaptation to cold environments and structure and bioenergetics are scarce. Their application potential has not yet been exploited fully for the benefit of mankind. Microbes with high potential are still waiting in the cold and harsh niches. This review attempts to summarize the developments in cold-active proteases, and strategies that can be adapted to search for more potent and versatile cold-active proteases to suit industrial requirements.

## 2. Microbes Producing Cold-Active Proteases

Cold-active proteases are mainly sourced from microorganisms from cold habitats such as arctic regions, polar regions, deep sea and glacier soils, glacier ice, permafrost, cold desert soil, sub-Antarctic sediments, sub-glacial water, alpine regions and other cold regions on earth. The potential of psychrophiles and enzymes produced by them have been reviewed from time to time [17,28,29,30,31]. Morita [32] defined psychrophiles based on their optimal growth temperature. Organisms growing optimally at about 15 °C or below with a maximum temperature of growth at about 20 °C and the ability to survive at 0 °C are known as psychrophiles. In contrast, psychrotolerant microbes generally have optimum and maximum temperatures of growth at 20 °C or above. Psychrotolerant microbes have an optimum growth temperature between 20 and 40 °C, but are also capable of growth at 0 °C [33].

Oceans cover more than 70% of Earth’s surface and it is a major ecosystem with an average temperature of around 5 °C, and hence, this is one of the habitats for psychrophiles. A diverse range of psychrophilic microorganism have been isolated from sea belonging to different microbial groups such as gram-negative (e.g., *Pseudoalteromonas, Moraxella, Psychrobacter, Polaromonas, Psychroflexus, etc.*) and gram-positive (e.g., *Arthrobacter, Bacillus, Micrococcus*] bacteria, archaea [e.g., *Methanogenium, Halorubrum*), yeasts (e.g., *Candida, Cryptococcus*) and fungi (e.g., *Penicillium, Cladosporium*).

Cold-active protease producing microorganisms have been isolated from different geographical regions such as *Azospirillum* sp. from mountain soil [34], *Bacillus licheniformis* from glacier soil [35] *Clostridium* sp. from Antarctic region [36], *Colwellia* sp. from sea ice [37] and sub-antarctic sediments [38], *Curtobacterium luteum* from glacier soil [39], *Exiguobacterium* sp. from cold desert soil [40], *Pedobacter cryoconitis* from glacier ice [41], *Penicillium chrysogenum* from cold marine environment [42], *Pseudomonas* sp. from deap sea [43], *Psychrobacter proteolyticus* from Antarctic krill *Euphasia superba* Dana [44], *Serratia* sp. from coastal water [45], *Vibrio* sp. from marine water [46] and *Xanthomonas maltophilia* from alpine environment [47]. Yu *et al.* [48] screened organisms from the sandy sediment of Nella Fjord, Eastern Antarctica [69°22′6″ S, 76°21′45″ E] for the cold-active hydrolytic enzymes. Out of 33 isolates screened, *Sulfitobacter* sp NF1-26, *Photobacterium* NF1-15, *Pseudomonas* NF1-39-1, *Shewanella* NF1-3, *Bizionia* NF1-21, *Flavobacterim* NF1-9, *Salinibacterium* NF2-5 were found to secrete proteolytic enzymes. While Kuddus *et al.* [49] isolated cold-active alkaline protease producing *Stenotrophomonas* sp. from the soil of Gangotri glacier (western Himalaya, India). Some microorganisms that are known to producecold-active alkaline proteases are listed in Table 1.

**Table 1:** Microorganisms producing cold-active alkaline protease.

S. No.	Organisms	Properties of the proteases			Reference
		Mol. weight (kDa)	T_Opt_ (°C)	pH_Opt._	
1	*Alcaligenes faecalis*	-	30	8.8	[50]
2	*Alkaliphilus transvaalensis*	30	40	12.6	[51]
3	*Alteromonas haloplanktis*	74–76	20	8–9	[52]
4	*Aspergillus ustus*	45	32	9	[53]
5	*Azospirillum* sp*.*	48.6	40	8.5	[34]
6	*Bacillus* sp.	-	30	9.6	[54]
7	*Bacillus* spp.	-	40	10.5–11	[55]
8	*Bacillus amyloliquefaciens* S94	45	-	10	[56]
9	*Bacillus cereus*	-	20	9	[57]
10	Bacillus *licheniformis* RKK-04	31	50	10	[58]
11	*Bacillus pumilus*	-	30	11.5	[59]
12	*Beauveria bassiana*	-	37	10	[60]
13	*Candida humicola*	-	37	10	[61]
14	*Clostridium* sp.	46	37	7	[36]
15	*Colwellia* sp.	60	35	8–9	[62]
16	*Colwellia psychrerythraea* strain 34H	71	19	6–8.5	[63]
17	*Curtobacterium luteum*	115	20	7	[39]
18	*Engyodontium album*	-	25	11	[64]
19	*Escherichia freundii*	55	25	10	[65]
20	*Exiguobacterium* sp.SKPB5	36	40	8	[40]
21	*Flavobacterium* YS-80	49	30	8–11	[66]
22	*Flavobacterium balustinum* P104	70	40	7–9	[67]
23	*Leucosporidium antarcticum* 171	34.4	30	8	[68]
24	*Pedobacter cryoconitis*,	27	40	8	[41]
25	*Penicillium chrysogenum* FS010	41	35	9	[42]
26	*Planomicrobium* sp. 547	-	35	9	[69]
27	*Pseudoalteromonas* sp. D12-004	34	35	7–8	[70]
28	*Pseudoalteromonas* sp. NJ276	28	30	8	[37]
29	*Pseudoalteromonas* sp. P96-47	-	20	8	[71]
30	*Pseudoalteromonas* sp. SM9913	65.84	25	9	[72]
31	*Pseudomonas* sp Ele-2	45	40	-	[73]
32	*Pseudomonas* sp.	-	20		[74]
33	*Pseudomonas strain* DY-A	-	40	10	[43]
34	*Pseudomonas aerugenosa* MTCC 7926	-	40	9	[75]
35	*Pseudomonas lundensis*	48	30	10.5	[76]
36	*Pseudomonas fluorescens*	-	35	5	[77]
37	*Pseudomonas fluorescens* 114*.*	47	35-40	8	[78]
38	*Pycnoporus cinnabarinus* ss3	-	30	4	[79]
39	*Roseobacter* sp. [MMD040]	-	37-40	8–9	[80]
40	*Serratia marcescens* AP3801	58	40	6.5–8.0	[81]
41	*Serratia marcescens* TS1	56	40	8	[82]
42	*Serratia proteamaculans* 94	50	4-30	8	[83]
43	*Shewanella* strain Ac10	44	5-15	9	[84]
44	*Stenotrophomonas* sp.	55	15	10	[85]
45	*Stenotrophomonas maltophilia* MTCC 7528	75	20	10	[49]
46	*Streptomyces* sp.	-	30	10	[86]
47	*Streptomyces alboniger*	-	37	9–11	[87]
48	*Teredinobacter turnirae*	-	25	7	[88]
49	*Trichoderma atroviride*	24	25	6.2	[89]
50	*Vibrio* sp.	35	40	8.5–9.0	[90]
51	*Vibrio* sp. PA-44	47	25	8.6	[46]

## 3. Classification of Proteases

According to the Enzyme Commission [EC] classification, proteases are members of the group 3 [Hydrolases], and sub-group 4 [hydrolyzing peptide bonds]. Proteases have been divided into two broad groups on the basis of their ability to hydrolyze N- or C- terminal peptide bonds [exopeptidases] or internal peptide bonds [endopeptidases]. Although exopeptidases are used in some commercial applications, endopeptidases are industrially more important than the former. Exopeptidases are subdivided as aminopeptidases that cleave the N-terminal peptide linkage and carboxypeptidases that cleave the C-terminal peptide bond.Several other features have also been used in classifying proteases into different groups such as occurrence of charged moieties at sites relative to susceptible bond [91], their pH optima [as acidic, neutral or alkaline], substrate specificity [collagenase, keratinase, elastase], or their homology to previously characterized proteases such as trypsin, pepsin and others [trypsin-like, pepsin-like]. Morihara [92] classified serine proteases as trypsin-like proteinases, alkaline proteinases, *Myxobacter* α-lytic proteinases and staphylococcal proteinases. Hartley *et al.* [93] classified endoproteases into four groups on the basis of their active site and sensitivity to various inhibitors. The properties of the enzymes are summarized in Table 2.

## 4. Optimization of Fermentation Conditions for Production of Cold-Active Proteases

Proteases produced by microorganisms are predominantly extracellular in nature and are greatly affected by nutritional and physicochemical factors. Optimization of different media components can greatly affect the production cost and can lead either to profit or loss in an industry based on production of bioactive compounds by microorganisms. Proper balance of various media components determines the utilization of each component. In order to have a cost effective method of enzyme production, optimization of various media components is needed. Importance of this step is revealed by the fact that 30%–40% of production cost of industrial enzymes is estimated to be the cost of the growth medium [94]. No single medium can be used for production of protease from different psychrotrophic microbes. Each microorganism has its own specific idiosyncratic, physicochemical and nutritional requirements for the production of maximum enzyme titer. Therefore, it is necessary to optimize the production conditions for the strain of interest. Protease production by psychrotrophic microorganisms is affected by media components such as changes in C/N ratio, presence or absence of some easily metabolizable sugars such as glucose and sucrose in the production medium. Casein was the best nitrogen source, but the presence of carbohydrates like glucose, sucrose and lactose led to catabolic repression of protease production in *Colwellia* sp. [37]. Metal ions in the surrounding environment affect the growth of the organisms. Some having positive effect and some inhibits the growth of the organism. It is critical to find out which metal ion supports both the growth of the organism under study and the protease production. In *Stenotrophomona* sp., the enzyme production was enhanced by Cu^2+^ [126.8%] and Cr^2+^ [134.6%], but Co^2+^ reduced it [43.5%]. The other heavy metals such as Hg^2+^, Cd^2+^ and Zn^2+^ had no significant effect [49]. Vazquez *et al.* [95] reported that increasing concentrations of calcium chloride [0 to 0.3 g l−1] in culture media enhanced protease production in *Stenotrophomonas maltophilia*; the highest titre was attained after 36 h of growth.

Most of the proteolytic enzymes are produced and secreted in late exponential growth phase [96]. *Stenotrophomona* sp. has been reported to secrete maximum enzyme at 120 h [49]. While the enzyme production by *Pedobacter cryoconitis* attained a peak in 72 h, and thereafter, there was plateau in enzyme production [41]*. Pseudomonas* sp. strain DY-A produced maximum protease after 30 h incubation [43]. In case of *Pseudomonas* sp. strain DY-A protease, 10 °C was found optimum both for growth and protease production. Temperature change to 25 °C reduced both the growth and protease production [43]. In *Stenotrophomona* sp., a high protease titre [56.2 U/ml] was attained at 20 °C. This observation suggested that high enzyme titers could be produced in the temperature range between 15 and 25 °C [49]. *Pedobacter cryoconitis* produced maximum enzyme at 15 °C, although 44% of the maximum enzyme titer was also attained at 1 °C [41].

**Table 2:** Classification and biochemical characteristics of endoproteases.

Endoprotease	EC No.	Mol. Mas Range (kDa)	pH_Opt._	T_Opt._ (°C)	Metal Ion Required	Active Site a Residues	Major Inhibitor(s)
Aspartic or Carboxyl proteases	3.4.23	30–45	3–5	40–55	Ca^2+^	Aspartate or cysteine	Pepstatin
Cysteine or thiol proteases	3.4.22	34–35	2–3	40–55	-	Aspartate or cysteine	Indoacetamide, *p*-CMB
Metallo- proteases	3.4.24	19–37	5–7	65–85	Zn^2+^, Ca^2+^	Phenylalanine or leucine	Chelating agents such as EDTA, EGTA
Serine proteases	3.4.21	18–35	6–11	50–70	Ca^2+^	Serine, histidine and aspartate	PMSF, DIFP, EDTA, soybean trypsin inhibitor, phosphate buffers, indole, phenol, triamino acetic acid

## 5. Purification of Cold-Active Proteases

Proteases have been screened and purified from different sources. Different strategies have been employed for purifying cold-active proteases from diverse sources. The purification strategies used for purifying cold-active proteases from different sources are presented in Table 3. Proteases secreted into the medium are first concentrated by using methods such as ultrafiltration [96,97,98], ammonium sulphate [36,39,42,43] or acetone precipitation [68,81]. A few methods involve use of PEG [72] and lyophilization [69]. After concentrating protein, further purification is achieved either by single technique or by combining two different methods. Ion exchange chromatography is a method of choice in maximum cases. DEAE [diethyl amino ethyl] and CM [carboxy methyl] group containing matrices are mainly used to which protein molecules get adsorbed and can be eluted either by pH change or change in ionic strength of the eluent buffer.

**Figure 1:**
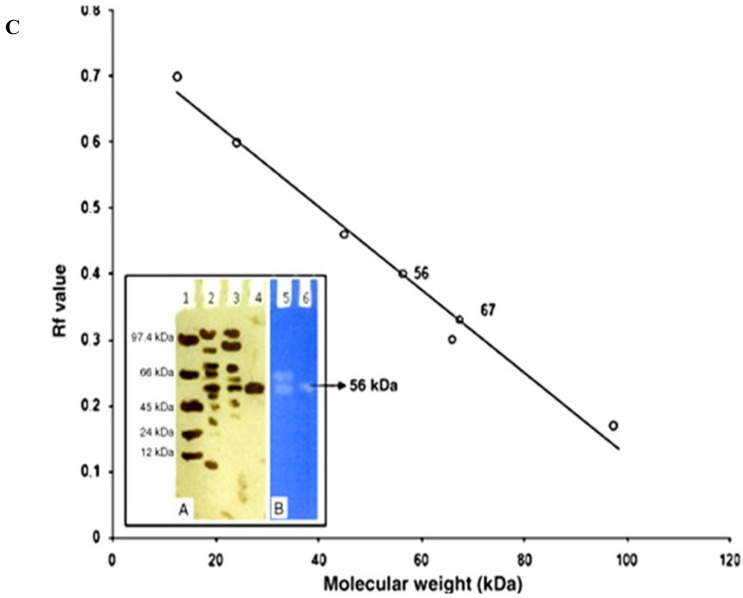
Native-PAGE of crude and purified protease from *P. aeruginosa* MCM B-327. (A) silver stained gel; (B) zymogram of protease with casein; (C) plot of R_f_ values *versus* standard molecular weights [99].

Affinity chromatography technique is also a successful method of purification but labile nature of affinity ligands and higher cost are limiting factors. Hydrophobic interaction chromatography [HIC] and gel filtration chromatography have also been used extensively for protease purification either at an early to middle stage or in the final stage. Sephacyl, Superdex, Superose and Topopearl gels are most commonly used for filtration purpose. Zambare *et al.* [99] used various chromatographic techniques to purify protease from *P. aeruginosa* MCM B-327 and determined its molecular weight (Figure 1).

**Table 3:** Strategies used for purification of cold-active proteases.

Protease Source	Protease Type	Concentration Method	Column Matrices	Fold Purification	References
*Alkaliphilus transvaalensis*	Serine protease	Amicon Ultra-15	DEAE Toyopearl 650M resin, CM-Toyopearl 650M	96	[58]
*Clostridium* species	Serine-type metalloenzyme	Ammonium sulfate precipitation	Sephadex G-100	12.7	[36]
*Colwellia psychrerythraea* strain 34H.	Aminopeptidase	-	Sepharose Q, Hydroxyapatite, Resource Q	460	[63]
*Curtobacterium luteum* MTCC 7529	Metalloprotease	Ammonium sulphate precipitation	DEAE- Cellulose	34.1	[39]
*Escherichia freundii,*	Neutral serine protease	Ammonium sulfate precipitate	CM-cellulose, DEAE-Sephadex A-50, Sephadex G-100	-	[65]
*Leucosporidium antarcticum* 171	Serine proteinase	Acetone precipitation	Sephadex G-75, Diethylaminoethyl-Sephacel, Sephacryl S-100	1,568	[68]
*Oerskovia xanthineolytica* TK-1	Serine protease	Ultrafiltration	Phenyl-Sepharose CL-4B, DEAE-Sephacel	39.6	[97]
*Pedobacter cryoconitis*	Metalloprotease	-	SP Sepharose, Syn-Chropak CM300	-	[39]
*Penicillium chrysogenum* FS010	Serine protease	Ammoniumsulfate precipitation	DEAE Sepharose, Sephadex G-100	103.2	[42]
*Planomicrobium* species	Serine protease	Ammonium sulfate precipitation, Lyophilization	DEAE-52	-	[69]
*Pseudoalteromonas* sp. NJ276	Serine protease	Ammonium sulfate precipitation	DEAE-Sephadex A50, Sephadex *G-75*	22.5	[37]
*Pseudoalteromonas* sp. SM9913	Serine protease.	Ammonium sulfate precipitation, PEG 2000.	Sephadex G100	-	[53]
*Pseudomonas aeruginosa* IFO 3455	Metalloprotease	-	QAE-agarose	-	[74]
*P. fluorescence* 114	Neutral metalloprotease	Ammoniumsulfate precipitation	DEAE Toyopearl 650 M, Superdex 200 HR 10/30	-	[78]
*Pseudomonas* strain DY-A	Serine protease	Ammonium sulfate precipitation,	DEAE Sepharose CL-6B, Sephadex G-100	84.2	[43]
*Serratia marcescens* AP3801	Metalloprotease	Ammonium sulfate precipitation	Sephacryl S-100, Q Sepharose	0.48	[62]
*S. marcescens* TS1.	Metalloprotease	Ammonium sulphateation, acetone precipitation	DEAE-cellulose	-	[82]
*S. proteamaculans*	Trypsin-like protease	Ultrafiltration	Q-Sepharose, BPTI-Sepharose	-	[98]
*S. proteamaculans*	Serine trypsin-like and Zn-dependent protease.	-	BPTI-Sepharose	-	[100]
*S. proteamaculans* 94	Cysteine protease	-	Arg-Silochrom Z-Gly- *DL*-Pro-Gly-Silochrom, Superise 12 HR 10/30 column	3433	[83]
*Shewanella* strain Ac10	Alkaline serine protease	-	Bacitracin-Sepharose column		[84]
*Stenotrophomonas maltophilia*	Serine proteases	Ultrafiltration	S-Sepharose	-	[101]
*Stenotrophomonas* sp.	Alkaline protease	Ammonium sulfate precipitation	DEAE-Sepharose	18.45	[85]
Marine psychrophilic strainPA-43	Serine peptidase	-	Q Sepharose, Sephacryl S-300, PBE 94	25.0	[102]
*Vibrio* sp. PA-44	subtilisin-like proteinase	Ammonium sulfate precipitation	N-carbobenzoxy-d-phenylalanyl-triethylenetetramine-Sepharose, phenyl-sepharose	-	[46]

## 6. Properties of Cold-Active Proteases

### 6.1. Temperature

Protease produced from *Stenotrophomonas maltophilia* MTCC 7528 is optimally active at 20 °C and the activity of the enzyme is retained even after repeated freez-thaw cycles [49]. M1 aminopeptidase (designated ColAP) produced by the marine psychrophilic bacterium *Colwellia psychrerythraea* strain 34H exhibited optimum activity at 19 °C [63]. Zhu *et al.* [42] reported optimal temperature for protease produced by *Penicillium chrysogenum* FS010 was 35 °C, about 10–15 °C lower than normally used industrial protease. The enzyme showed relatively high activity between15 and 35 °C. It retained 41% of proteolytic activity at 0 °C. Protease produced by *Pedobacter cryoconitis* showed optimum temperature for activity was 40 °C. Activity was significantly reduced at 50 °C, and total inactivation occurred at 60 °C. High activity (28%–79% of the maximum activity) was detected at 20–35 °C [41]. 

### 6.2. pH

The activity of any enzyme is greatly affected by the pH of the reaction mixture. Each enzyme has its own optimum pH at which is shows maximum activity. Protease fall into different classes based on their pH optima. An alkaline protease isolated from *Stenotrophomonas maltophilia* MTCC 7528 has been shown to be optimally active at pH10 [49]. Protease produced by Pseudoalteromonas sp. NJ276 has optimum enzyme activity at pH 8.0. About 31 and 38% of its optimum activity is detectable at pH 5.0 and 11.0, respectively [35]. Protease of *Pseudomonas* sp. strain DY-A showed a broad pH profile (pH 6.0–12.0) for casein hydrolysis and highest activity between pH 8.0 and 10.0. The highest stability of this protease was recorded at pH 10.0 [43].

### 6.3. Metal Ions

Cold-active protease from *Pseudoalteromonas* sp. NJ276 was partially inhibited by metals such as Mg^2+^, Ca^2+^, Cu^2+^, Zn^2+^, Ba^2+^, Fe^2+^, Pb^2+^ and Mn^2+^. The enzyme was stable after incubation for 1 h in the presence of 2M NaCl, moreover, 56.5% of the maximum activity was detected in the presence of high-salt concentrations (up to 3M NaCl) [37]. Zeng *et al.* [43] reported that total activity of protease from *Pseudomonas* sp. strain DY-A production medium was increased by 30% in the presence of Ca^2+^ and Mg^2+^ (10 mM). These metal ions enhanced the enzymatic activity slightly (8%) and had an important role in enzyme stability. Among the cations tested, Co^2+^, Cu^2+^ and Zn^2+^ inhibited the enzymatic activity, while Fe^3+^, Mn^2+^, K^+^, Li^+^, Hg^+^, Ag^+^ had no observable effect on enzymatic activity [43]. *Penicillium chrysogenum* FS010 protease activity was increased by the addition of Ca^2+^, Na^+^, Mg^2+^, K^+^, NH4 ^+^, while Cu^2+^, Co^2+^, Fe^3+^ and EDTA inhibited the enzyme activity. The inhibitory effects of Fe^3+^ and EDTA were the strongest [43]. Cu^2+^ and Fe^3+^ showed strong inhibitory effect on Ps5 metalloprotease of *Pseudomonas lundensis,* while Co^2+^, Fe^2+^, Mn^2+^, Al^3+^ reduced enzyme activity to 32%–14% [76]. Yang *et al.* [76] suggested that there could be a new catalytic pathway for reaction mechanism of Ps5 protease as contrary to other metalloproteases it showed inhibition by Zn^2+^. The activity of ColAP was strongly inhibited by Zn^2+^ and Mn^2+^, while Ca^2+^ was slightly inhibitory, Mg^2+^ stimulated activity at 10 mM or higher, which is almost equivalent to the concentration found in seawater [63].

### 6.4. Effect of Inhibitors and Other Reagents

Cold-active protease of *Pseudoalteromonas* sp. NJ276 was inhibited by phenylmethylsulfonylfluoride [PMSF], sodium dodecyl sulfate [SDS], urea, thiourea, dithiothreitol [DTT], ethylenediaminetetraacetic acid [EDTA] and ethylene glycol tetraacetic acid [EGTA] but cystein protease inhibitor E-64 had no effect on the activity [37]. *Pseudomonas* sp. strain DY-A protease was completely inhibited by 1 mM of diisopropyl fluorophosphate (DFP), PMSF and 4-(2-Aminoethyl) benzenesulfonyl fluoride hydrochloride [AEBSF] which indicates that it was a serine protease. The enzyme was found resistant to thiol reducing agents such as DTT (10 mM) and β-mercaptoethanol [β-ME] (5%), suggesting that disulfide bonds were not involved in preserving proteolytic activity. The enzyme was found sensitive to urea (4 M), SDS (1%) and guanidine-HCl (1 M), indicating that hydrogen bonds played an important role in preserving the enzyme activity [43]. Seventy percent activity of cold-active metalloprotease Ps5 of *Pseudomonas lundensis* HW08 was inhibited by 10mM PMSF [76], indicating that it was a serine protease. Only 40% of the maximum activity was left after treatment with 1mM EDTA while EGTA had no significant effect on activity of Ps5 protease. Ps5 protease exhibited an interesting property of stimulation of activity 124% at 1.0% of H_2_O_2_, and retained 73% activity on increasing H_2_O_2_ concentration to 10%. Moreover 10% urea stimulated enzyme activity, but it lost the activity completely in the presence of anionic detergent SDS even at 1%. Hustan *et al.* [63] reported that the activity of ColAP was not affected significantly by PMSF, but inhibited by 10 mM DTT (a reducing agent) and EDTA (a metal-chelating agent). 

### 6.5. Catalytic Efficiencies

Although the environmental and physiological effects on microorganisms dwelling in cold environments have been understood fairly well, the mechanistic details that allow enzymatic reactions in the cold niches have not been understood adequately. Cold temperatures lead to exponential decreases in rates of chemical reactions, as clear by the Arrhenius equation, and also lead to increase in the compact folding of proteins, and thus restricting the conformational ease needed for catalysis [102]. Despite these setbacks, cold-active enzymes have evolved in nature. In contrast to mesophilic enzymes, these enzymes exhibit three general distinguishing features: a higher specific activity [*k*_cat_] or catalytic efficiency [*i.e.*, *k*_cat_/*K*_m_] at temperatures between 0 and 30 °C, a lower optimal temperature for activity, and reduced stability due to temperature rise and denaturating agents [103]. Kinetic parameters like *K*_m_ and *k*_cat_ have been studied in several psychrophilic enzymes and compared with their mesophilic and thermophilic counterparts [14,103]. Enzymes usually buried in huge amount of substrate tend to optimize their *k*_cat_ rather than *K*_m_ [14] for efficient functioning at low temperatures. Cold-active protease from *Clostridium* species isolated from Schirmacher oasis, Antarctica showed an increase in *K*_m_ with decrease in temperature which appears to be a characteristic that indicates a weak substrate binding which in turn lowers the activation energy [36]. This strategy to improve catalytic efficiency at low temperature has been adopted by many cold-active enzymes from Antarctic bacteria including alpha amylase from *Alteromonas haloplanctis* [104] and β-lactamase from *Psychrobacter immobilis* [14]. The *Clostridium* protease also exhibited a *Q*10 value (1.91) which is much lower than that observed for most mesophilic enzymes, which are in the order of 2 to 3 [14]. True psychrophilic enzymes are more flexible in structure and are invariably thermolabile. Despite displaying adaptational features of a cold-active enzyme like low *Q*10 value and increased *K*_m_ at low temperatures, protease from *Clostridium* has been moderately thermostable [36].

The Michaelis-Menten constant [*K*_m_] and catalytic efficiency [*k*_cat_/*K*_m_] values for protease from *Pseudoalteromonas* sp. NJ276 were 0.41 mM and 45 s^−1^mM^−1^, respectively at 35 °C. The enzyme retained 54% of *k*_cat_ and *k*_cat_/*K*_m_ at 0 °C. The activation energy of the protease was 34.8 kJ mol^−1^ [37].

Huston *el al.* [63] reported the highest specific activity [*k*_cat_] for *Colwellia psychrerythraea* strain 34H cold-active protease (ColAP) at 19 °C. The highest *k*_cat_/*K*_m_ value for ColAP was also recorded at 19 °C (5.0 s^−1^mM^−1^), and 44% of this was retained at 9 °C which is the optimum growth temperature for the strain 34H.

### 6.6. Substrate Spectrum

Proteases generally exhibit broad substrate specificity and are found active against both synthetic substrates and native proteins. Zeng *et al.* [43] reported *Pseudomonas* sp. strain DY-A protease displayed high activity towards N-succinyl-Ala-Ala-Pro-Phe-p-nitroanilide and N-succinyl-Ala-Ala-Pro-Leu-p-nitroanilide , which are well-known substrates for chymotrypsin but showed no activity towards N-Succinyl-Ala-Ala-Pro-Asp-p-nitroanilide, N-Succinyl-Ala-Ala-Ala-p-nitroanilide, N-Succinyl-Gly-Phe-p-nitroanilide. MCP-3 protease from *Pseudoalteromonas* sp. SM9913 displayed a broad substrate specificity and hydrolysed AAPF [N-Succinyl-Ala-Ala-Pro-Phe-p-nitroanilide] and partially hydrolyzed AAPL[N-Succinyl-Ala-Ala-Pro-Leu-p-nitroanilide], AAPK [N-Succinyl-Ala-Ala-Pro-Lys-p-nitroanilide], AAPR [N-Succinyl-Ala-Ala-Pro-Arg-p-nitroanilide], FAAF [N-Succinyl-Phe-Ala-Ala-Phe-p-nitroanilide] and FVR [N-Benzoyl-Phe-Val-Arg-p-nitroanilide] [105]. The protease from *Pseudomonas fluorescens* hydrolyzed various proteins with preference for milk proteins, caseins, as substrates [106]. Urea-Hb (urea-denatured haemoglobin) is the preferred substrate of the Antarctic yeast *Leucosporidium antarcticum* 171 proteinase lap2. The activity against native Hb and casein was found 60%–70% lower. The subtilase showed poor activity on elastin [68]. Psychrotrops have been reported to preferentially use synthetic substrates with proline at position P2 and an aromatic residue rather than an aliphatic residue at position P1 [63,102,107]. Protease from *Shewanella* strain PA- 43 displayed high activity towards N-succinyl AAPF p-nitroanilide. N-succinyl AAPL p-nitroanilide was also a relatively good substrate but showed reduced activity for N-succinyl AAVA p-nitroanilide. N-succinyl GGF p-nitroanilide, N-succinyl AAA p-nitroanilide and N-succinyl AAPD p-nitroanilide were poor substrates, and there was no activity with N-succinyl AAV p-nitroanilide, N-succinyl GFG p-nitroanilide, N-succinyl L-Phe p-nitroanilide, or N-benzoyl DL-Arg p-nitroanilide [102]. Acidic protease from psychrotrophic yeast *Candida humicola* hydrolyzed poly-L-Ala, poly-L-Ser, poly-L-Phe, and poly-L-Glu but was more active on native proteins such as BSA, casein, gelatin, and melittin [61]. *Flavobacterium balustinum* protease displayed endoprotease activity with N Suc- AAPL-p-nitroanilides, whereas, there was no exoprotease activity of protease when L-Ala-pNA and L-Phe-pNA was used as substrate [50]. As proteases from psychrotrophs have been found to exhibit wide range of substrate specificity, they can be utilized for bioremediation of industrial and domestic waste at ambient temperature.

## 7. Cloning and Expression of Cold-Active Proteases

Recombinant DNA technology has revolutionized the enzyme industry by providing a means for making the enzyme production process very economical. Till date very few attempts have been made in cloning of cold-active proteases and expression in heterologous or homologous hosts. Ni *et al.* [108] reported cloning of a 1248 bp long alkaline protease gene ORF (encoding a 42.9 kDa protein) from marine yeast *Aureobasidium pullulans* HN2-3 into surface display vector pINA1317-YlCWP110. This gene (ALP2) was heterologously expressed in cells of *Yarrowia lipolytica*. The recombinant *Y. lipolytica* cells displayed protease on its surface and were able to produce bioactive peptides from different sources of proteins. Khairullin *et al.* [98] sequenced and cloned a 78kDa novel trypsin like protease (PSP) from *Serratia proteamaculans.* The protease was cloned in *E. coli* expression vector pET23b (+) and trsansformed into *E. coli* BL21 [DE3] expression strain. Recombinant protein was purified using Ni^2+^-NTA agarose column. The yield of expressed His6-PSP was 150 mg from 100 g of biomass. Yan *et al.* [105] cloned cold-adapted halophilic proteases from deep-sea psychrotolerant bacterium *Pseudoalteromonas* sp. SM9913. Yan *et al.* cloned the protease gene into pET22b (+) and expressed the gene as active protein in *E. coli* BL21 [DE3] cells. The recombinant protein was purified from fermentation broth as a multidomain protein containing one catalytic domain and two PPC domains which were further characterized using purified recombinant protein. Wintrode *et al.* [109] used cloning and expression technique to study the reversal of the properties of a mesophilic subtilisin like protease from *Bacillus sphaericus* to a protease resembling more to a psychrophilic protease. Directed laboratory evolution approach used by Wintrode resulted in generation of a protease which showed rate constant [*k*_cat_] at 10 °C 6.6 times and a catalytic efficiency [*k*_cat_/*K*_m_] 9.6 times that of wild type. Its half-life at 70 °C is 3.3 times less than wild type. Wintrode *et al.* (109) used *E. coli- Bacillus* shuttle vector pSPH2R and a protease deficient *Bacillus* strain DB 428 for their study. DNA library was prepared in *E. coli* HB101 cells and then transformation of *Bacillus* competent cells was performed. Taguchi *et al.* [110] also took assistance of cloning and expression technology to improve psychrophilic features of a cold-active protease. The mutant subtilisin m-63 showed *k*_cat_/*K*_m_ value 100% higher than that of the wild type at 10 °C when *N*-succinyl-L-Ala- L-Ala-L-Pro-L-Phe-p-nitroanilide was used as a substrate. This cold adaptation resulted due to three mutations, Val to Ile at position 72 [V72I], Ala to Thr at position 92 [A92T], and Gly to Asp at position 131 [G131D], and it was observed that an enhancement in substrate affinity was mostly responsible for the increased activity. Taguchi *et al.* [110] used pUC18 and pHY300PLK expression vectors for *E. coli* and *Bacillus* respectively and *E. coli* JM 109 and *Bacillus subtilis* UOT0999 as expression hosts. Kulakova *et al.* [84] prepared genomic library using genomic DNA of *Shewanella* sp. strain Ac10 in pUC118 and then selected positive clones expressing serine alkaline protease [SapSh]. Positive clones were used for subsequent retrieval of protease ORF from the pUC118 clone and then sub-cloning in pET21a for cloning the SapSh under T7 lac promoter. Recombinant protein was functionally active but shorter in size (44 kDa) than expected (85 kDa) which indicated removal of some protein sequence during protein processing. Sheng *et al.* [69] also reported cloning of alkaline protease gene from psychrophilic *Planomicrobium* sp. 547 into pTA2 vector. It has been felt since long that a different expression host should be developed for expressing cold-active proteins, since this will assist the proteins is several ways like proper folding and thus retaining activity. Parrilli *et al.* [111] developed *Pseudoalteromonas haloplanktis* TAC125 as a versatile psychrophilic host for recombinant protein production by disrupting its *gspE* gene. Other psychrophilic hosts should be generated so that a diverse array of cold-active proteins along with industrially important protease can be heterologously expressed.

## 8. Crystal Structure of Cold-Active Proteases

X-ray crystallography has revolutionized the study of proteins. So far structure-function relationship of many enzymes has been deduced using this technique. Understanding of tertiary structure of enzymes and spatial arrangement of catalytically important functional groups must be known in order to get information about possible changes in enzyme structure resulting from binding of substrate, products, stabilizers, inhibitors or effector molecules. 3D structure of proteases can provide an insight into the mechanism of the enzymatic action and also provide a template for the design of novel drugs if the studied protease is involved in pathogenesis, e.g., viral ptoreases. Crystallographic studies of proteins can also assist in understanding molecular basis of structure-environment adaptation relationships. Thus proteins in cold environments can be understood in further detail by this technique. Comparative investigations of numerous protein models and crystal structures revealed that cold-adapted enzymes tend to exhibit an attenuation of the strength and number of structural factors known to stabilize protein molecules [112].

Recently Almog *et al.* [113] reported that the calcium-loaded state is not responsible for the cold adaptation of psychrophilic cold-adapted subtilisin S41 from Antarctic *Bacilllus* sp. TA41. This conclusion was reached based on comparison of crystal structure of S41 with a mesophilic subtilisin Sph from *Bacillus sphaericus* 22973. These two subtilisins were highly similar in their calcium binding mode but differed in cold adaptability.

**Figure 2:**
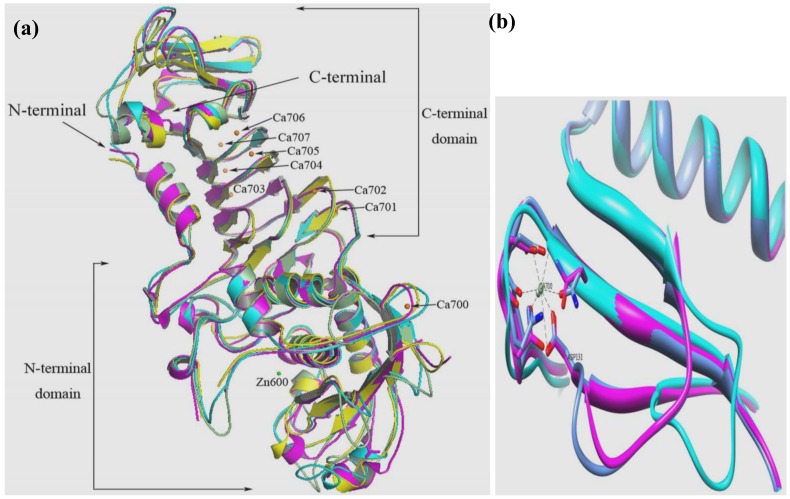
**(a)** superimposed image of MP [in yellow] with PAP forms 2 [in magenta color] and AP [in cyan] and SMP [in pale green]. Zn and Ca ions shown in figure are from the structure of MP. **(b)** additional N-terminal Ca^2+^ binding site is shown in superimposed image of MP [in cornflower blue] with PAP form 1 [in purple] and AP [in cyan] demonstrating a stabilized loop formation shaped in MP. Amino acids of MP which are coordinating to the Ca ions are depicted as sticks [66].

 Many cold-active enzymes have been studied so far using crystallographic technique. Some of them include *Arthrobacter* β-galactosidase [114], Lipase [115] of *Moraxella* TA144 a strain from Antarctica, Lipase from *Psychrobactor immobilis* [116]. Dong *et al.* [117] solved crystal structure of subtilisin like and psychrophilic protease Apa1 from Antarctic *Pseudoalteromonas* sp. strain AS11. An aminopeptidase from *Aeromonas proteolytica* was solved at 1.8 Å by Chevrier *et al.* [118]. Zhang *et al.* [119] solved the crystal structure of psychrophilic protease from *Flavobacterium* YS-80 at 2 Å. The marine protease from *Flavobacterium* acquires a two domain structure. N-terminal domain includes amino acid residues 37–264 and C-terminal comprise of residues from 265 to 480. Zhang *et al.* (66) compared the structural feature of the marine protease MP of *Flavobacterium* YS-80 with another psychrophilic protease PAP from Antarctic *Pseudomonas* species and mesophilic counterpart AP from *Pseudomonas aerugenosa* and SMP from *S. marcescens* (Figure 2). It was found that the Zn^2+^-Tyr-OH bond in PAP is more flexible in order to facilitate substrate accessibility and to maintain its activity in very low temperatures. Marine protease MP contains seven glycine residues which are not present in AP. Glycine is responsible for flexibility to the protein thus indicating that MP is more flexible in nature than AP [19]. Eight Ca^2+^ ions and one Zn^2+^ ions have been positioned in the electron density map. The MP has a comparable overall structure to PAP, AP and SMP with as it has a two domain structure as described earlier. After overlapping the overall structure of MP with PAP, AP and SMP using all the Ca atoms, the main-chain RMSDs were 0.87 Å [with PAP form1], 1.08 Å [with PAP form2], 1.03 Å [with PAP form3], 1.00 Å [with AP] and 1.28 Å [with SMP] [66]. 

Papaleo *et al.* [119] studied seven mutant enzyme structures generated by digital effects to know whether substitution of few chosen amino acid residue confer a change in overall shift in optimum temperature or thermal stability of cold-adapted α-amylase. The mutation of a few residues alone would not change the thermal behavior of the particular enzyme, and other features also must be addressed to alter the thermal stability of the protein. Such a study can also be carried out for protease also to deduce if thermal stability of the enzyme can be altered. The limited knowledge of the protein structure is quite inadequate to reach the any thumb rule for deciding temperature range of enzymes.

However a general model proposed to explain higher activity at low temperature is that the enzyme possesses a more flexible conformation, metaphorically like an open hand, than their mesophilic and thermophilic counterparts. As a result of this increased flexibility, protein would be thermolabile often observed with cold-active enzymes [120]. In contrast, thermostable enzymes have more rigid and compact conformation more like a fist protecting them against destabilizing forces occurring at higher temperatures. A goal in comparing proteins from extremophiles is to test this and other proposed models for confirming whether such changes result in flexibility. Directed evolution studies have recently indicated that there is not a direct correlation between increased activity at low temperature and decreased thermostability [121]. In order to understand structural basis of cold adaptation, more crystal structures of similar mesophilic and thermophilic counterparts, along with the rationalized mutation studies are required. The most frequently reported structural differences between cold-adapted psychrozymes and their thermophilic and mesophilic counterparts involve interactions like fewer intra- or inter- subunit salt-bridges, loosely held hydrophobic packing in the protein core, longer surface loops and fewer prolines in such loops, increased number of glycine clusters, reduced number of arginines, improved solvent interactions through additional surface (mostly negative) charges, increased solvent exposure of apolar surface and a better accessibility of the active site [122].

## 9. Cold Environment Metagenomics: Tapping Biodiversity

Geographical regions with low temperature harbor psychrophiles and psychrotolerants. Diverse environments form extremely diverse niches, and the microorganisms are exposed to various extremes like pressure, temperature, nutrient availability and light. These organisms are a treasure of potentially unique biochemical and molecular profiles that might have the enzyme or molecule of enormous biotechnological interest and industrial application. The microbial enzymes from such environments are expected to have quite diverse biochemical and molecular properties. Isolation of both the microbe of interest and the molecule or enzyme of interest from these niches encounters obstacles mainly due to two reasons: first, despite the recent advances in the development of new culturing methodologies, most extremophiles could not be cultured using available technologies, and second, the problem of very low amount of biomass and thus the yield of DNA is very low for molecular analysis. Environmental genomics provides an answer for exploitation of the wealth offered by nature in extreme environments. Basic steps in this approach involve sample collection from the niche of interest such as cold environment and then this sample is processed for isolation of total environmental DNA. This environmental DNA is used either for cloning into suitable vector for genomic library construction or directly for sequencing or amplification using universal primers. These libraries are screened for the presence of enzymes of interest or for biomolecules of interest. The use of high throughput screening techniques and robotic systems make the screening process much faster and useful as large number of clones and libraries can be screened in relatively shorter time. 

Environmental genomics approach has been used for isolating many cold-adapted enzymes like lipases [123,124], cellulases [125], amylases [126], xylanases [127] from the microflora existing in the cold environments. The isolation and characterization of various novel cold-adapted enzymes highlights and supports the potential of the cold environment metagenomics in future for the discovery of psychrophilic proteases too. Berlemont *et al.* [128] isolated three proteases along with other commercially important enzymes from Antarctic soil metagenome. This approach will accelerate the biotechnological exploitation of microbial diversity present in cold environments. 

## 10. Enhancing Thermo-Stability of Cold-Active Proteases

The cold-active proteases from different microorganisms vary in their thermostability and alkalistability [41,43,73,65,63,101,105,129,130,131]. High proteolytic activity at lower temperatures shown by cold-active proteases is important in the commercial usage of proteases, but their low thermal stability is a common drawback that hinders their use in industries. To overcome this problem, various strategies have been used, among which reinforcement of the overall rigidity of the enzyme structure by increasing the number of disulfide bridges, intra-molecular salt bridges and shortening the length of loop regions are most commonly used [132,133]. Several ideas have been put forward for explaining the thermostability of proteins. In addition to providing insight into structural modifications, protein fluctuations can provide a mechanism of thermal stability too [134]. Experimental techniques such as nuclear magnetic resonance (NMR) [135,136], neutron diffraction methods [137] and theoretical approaches based on computer simulations on protein dynamics in solution [138,139] have supported the proposed idea. Molecular dynamics simulation has also been used to provide detailed atomic models of the protein stability and dynamics [140,141]. Attempts have been made to tailor the psychrophilic enzymes to have properties of industrial interest such as increased thermostability, tolerance to bleaches and detergents and to different organic solvents so that the proteins can become process friendly. The protein engineering has been used to alter the properties of the proteins by making changes in their primary structures. Mainly two engineering techniques have been used in attempts to create thermostable proteases: one is random mutagenesis and second is site-directed mutagenesis [SDM]. Pantoliano *et al.* [142] reported the improved thermostability and extreme alkalinity of subtilisin BPN by substitution of six individual amino acids [N218S, G169A, Y217K, M50F, Q206C, N76D]. The inactivation rate decreased several times as compared to the wild type BPN subtilisin. Strausberg *et al.* [143] reported 1000 times increase in t_1/2_ of subtilisin BPN of *B. subtilis* by loop removal, cassette mutagenesis and screening procedure. Shao *et al.* [144] reported 8-fold increase in t_1/2_ of subtilisin E by random priming and screening methods. 

A mutant subtilisin E with enhanced thermostability at 60 °C was generated using SDM [145]. The thermostability and activity of subtilisin-like serine proteinase [VPR] had been improved by SDM approach [146]. Such studies clearly indicate that it is possible to improve one character [activity/stability] without affecting the other. Narinx *et al.* [147] performed SDM for introduction of an additional salt bridge, disulfide bonds, and increasing the affinity of the enzyme for calcium, and found that stability of the molecular structure was achieved by a modification of a calcium ligand T85D. The mutated enzyme was thermostable like mesophilic subtilisin.

Directed evolution of proteins involves recombinant DNA techniques such as DNA shuffling, random priming recombination and the staggered extension process [StEP]. Zhao and Arnold [148] reported increase in thermostability of subtilisin E by converting it to thermitase using directed evolution. 

While studying the effect of trimethylamine N-oxide (TMAO) on the structure, activity, and stability of a psychrophilic protease (deseasin MCP-01), He *et al.* [149] suggested the possibility of using TMAO as an effective stabilizer to enhance the thermostability of a cold-adapted enzyme without compromising with its psychrophilic characters such as its overall structural flexibility and high catalytic efficiency at low temperature. The isolation temperature plays an important role in determining the cold adaptability of the enzyme of interest, as isolation temperatures is found to affect the enzyme properties. Vazquez and Mac Cormack [150] reported that the lower the strain isolation temperatures better the cold-adapted proteases in terms of optimal temperature and activation energy. Thus by using the recombineering and classical methods, thermostability of cold-active proteases can be improved.

## 11. Applications of Cold-Active Proteases

Economic benefits can be achieved by using cold-active proteases as they allow working at low temperatures even in an industrial scale. For example instead of heating and bringing the temperature during the industrial peeling process of leather by conventional protease from mesophilic or thermophilic microbes, the process can be performed at the temperature of tap water by using cold-active proteases. With the use of cold-active protease, energy saving is possible. 

**Figure 3:**
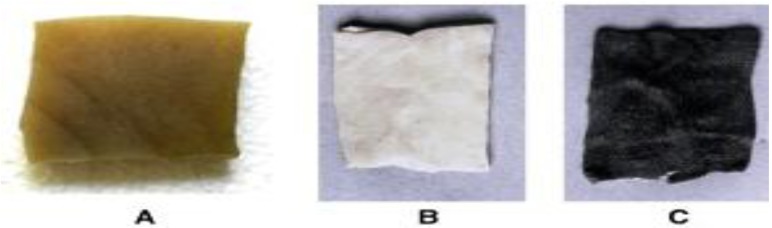
Buffalo hide dehairing by PA02 protease of *P. aeruginosa* MCM B-327. (a) chemical treatment, (**b**) crude enzyme treatment, (**c**) control-water treatment [99].

Proteases as a group found application in various fields such as baking, brewing, cheese making, in preparation of protein concentrates, leather industry, silk degumming, detergent industry, pharmaceutical industry, bioremediation, silver recovery from X-ray film and photographic industry are few to name the areas. The cold-active proteases find application in household processes, where they can be used for removal of macromolecular stains from fabrics along with other detergent components. As the whole process would be done at low temperature, the colors of the clothes will remain protected exposure to higher temperature. The treatment of wool and silk by protease can bring new and unique finishing to the surface of wool and silk fibers. Nowadays in textile industry, the synthetic fiber is being used. Some of the synthetic fibers cannot tolerate temperatures above 50–60 °C, and hence, require varied washing procedures [151]. During the past few years, a trend of lower washing temperature has gained popularity. Cold-active protease from *B. subtilis* showed stability in the presence of SDS and exhibited enhanced activity in Tween 80 and Wheel detergent, pH and detergent compatibility at low temperature, and thus, suits application in detergent formulation [35]. Protease from *Bacillus* sp.158 has found application in contact lens cleaning, thus increasing the transmittance of the lenses [152]. The protease of *P. aeruginosa* MCM B-327 was found to be useful in dehairing hides (Figure 3) [99].

In the food industry, the property of having high catalytic activity at low-temperature allows transformation of heat labile products. They can be used in processes such as fermentation of fish or soy sauce with no spoilage and alterations in flavor and nutritional value. Cold-active proteases along with lipases can be used as rennet substitutes to accelerate the ripening of slow-ripening cheeses. Additionally cold-active proteases can find utility in softening and taste development of frozen or refrigerated meat products. Apart from this, thermal lability of such proteases can result in rapid inactivation by mild heat treatment [30]. This feature will prove beneficial in preserving the quality in the food industry.

Cold-adapted or low temperature tolerant enzymes suit well in waste management in cold environments, where the degradation capabilities of endogenous microflora are reduced due to low temperatures. Cold-adapted proteases thus can be used to optimize present day industrial processes and for developing future technologies with less energy inputs and process cost by removing the cost of heat inactivation step [28,30].

## 12. Conclusions and Future Perspectives

A wide range of microorganisms from diverse habitats, permanently cold as well as those exposed to cold during a part of the year, are known to produce cold-active proteases. Metagenomic culture-independent approaches have also been initiated for obtaining novel cold-active biocatalysts including proteases. A few attempts have been made to engineer and manipulate the cold-active proteases, but much success has not yet been achieved. Cloning of genes encoding cold-active protease from the wild strains and their over expression in suitable hosts is another area of research for cost effective production of these enzymes. The field of cold-active protease research is still wide open and expected to achieve spectacular success in the nearest future. 
